# Epithelial Mesenchymal Transition: a double-edged sword

**DOI:** 10.1186/s40169-015-0055-4

**Published:** 2015-04-14

**Authors:** Guislaine Barriere, Pietro Fici, Giulia Gallerani, Francesco Fabbri, Michel Rigaud

**Affiliations:** Istituto Scientifico Romagnolo per lo Studio e la Cura dei Tumori (IRST) IRCCS, Via Piero Maroncelli 40, 47014 Meldola, FC Italy

**Keywords:** EMT, Cancer, Embryogenesis, Fibrosis, Wound healing

## Abstract

Epithelial mesenchymal transition (EMT) is a physiological process necessary to normal embryologic development. However in genesis of pathological situations, this transition can be perverted and signaling pathways have different regulations from those of normal physiology. In cancer invasion, such a mechanism leads to generation of circulating tumor cells. Epithelial cancer cells become motile mesenchymal cells able to shed from the primary tumor and enter in the blood circulation. This is the major part of the invasive way of cancer. EMT is also implicated in chronic diseases like fibrosis and particularly renal fibrosis. In adult organisms, healing is based on EMT which is beneficial to repair wounds even if it can sometimes exceed its goal and elicit fibrosis. In this review, we delineate the clinical significance of EMT in both physiological and pathological circumstances.

## Introduction

Epithelial tissues are the basis of most complex organs. Apical-basal polarity, cell-cell junctions allow tight physical coupling and enable epithelial cells to form sheet structures of generally crystalline order [1,2]. Epithelial sheets can actively migrate during physiological or pathological processes: embryogenesis, wound healing and cancer development. Over the course of these events, individual mesenchymal cells undergo a dispersion supported by an epithelial mesenchymal transition (EMT). EMT drives cells between two opposite flexible states: epithelial or mesenchymal. Such bold phenotypes are not an absolute rule. Rather than being all-or-nothing EMT is a fine-tuned manner regulated transition for each individual cancer cells. If EMT is a pathological phenomenon in cancer, its embryonic mirror picture will lead to organogenesis, necessary to living beings development. Moreover EMT occurs during the wound healing process. The latter leads when deregulated to fibrosis. In this review we will consider EMT through embryogenesis, in pathological situations like wound healing, fibrosis and finally in oncologic relapses and metastasis. We shall underline what could be the role of EMT in clinical applications.

## Review

EMT appears to occur in developmental steps during neural crest formation, gastrulation in the primitive streak somite decondensation, cardiac valve formation and other embryological events [3]. Common signaling pathways lead to delamination and migration of epithelial cells. EMT throughout embryogenesis highlights and provides important clues to explain abnormal development or loss of the differentiated state. Many signaling proteins and transcription factors are involved in EMT. Epithelia layered on extracellular matrix (ECM) are separated from it by basal membrane. Their cells have an apical-basal polarity and they are linked together by junctions. The latter are made of specific proteins which build adherens junctions and desmosomes. At the top lateral zones, tight junctions provide sealed connexions. Cells are also related one to another by gap junctions which furthermore support metabolism exchanges [4]. E-cadherin is a typical cadherin implicated in cell adherens junctions. Cadherins are linked to the cortical actin cytoskeleton via catenins. Desmosomes contribute to adhesion. Their structure includes cadherins, desmocollins and desmogleins which interact with cytokeratins through plakoglobulin and plakophilins. Integrins of hemidesmosomes account for basal adhesion [5-7]. The EMT event is characterized by up or down regulation of many proteins that support the epithelial architecture. The regulation is dependent on a web of chemical pathways specific to the type of EMT and tissues. EMT can be classified according to the circumstances of its occurrence. In embryology the phenomenon is called Type 1 EMT [8]. In the context of cancer, EMT is subverted and termed Type 3 EMT. Type 2 EMT leads to generation of new fibroblasts particularly in the field of renal injury [9].

### EMT and embryogenesis

EMT is a normal process necessary to development of the body plan: histogenesis and organogenesis. It was known from embryologists studies as soon as 1879 [10] and its revival was highlighted by publications of Greenburg and Hay [11,12]. Gastrulation, a reorganization of single layed embryo into three layers formation was first described by Trelstad et al. They described this phenomenon in chick embryo [13]. From these results, there were exponential publications on this topic.

From this pioneer work many research developments were led on role of EMT in gastrulation, heart formation (including endothelial mesenchymal transition), neural crest. They were realized by using different animal models: drosophila, sea urchin, chicken and mouse embryos. One the best embryological example of EMT is described in mouse embryo gastrulation. The latter is characterized by down regulation of E-cadherin. This protein is controlled at the transcriptional level by Snail1 and at posttranscriptional level by P38 interacting protein [14-16]. Among typical events of EMT, like involution (partial EMT), ingression is a process that allows single cells to delaminate and migrate into the sub-epiblast territory. At the cellular level, it can be explained by a cascade of biochemical reactions. When a cell with intact junctional complexes and epithelial polarity is submitted to EMT, growth factors activate membrane receptors in such a manner that actin cytoskeleton is remodeled and apical-basal polarity lost. Then DDR1 complex is able to activate RhoE resulting in actomyosin contractility weakness [17]. Non canonical pathways are triggered by tight junctions TGFβ receptors leading to ubiquitynilation and degradation of RhoA that destabilize cortical actin microfilaments. Then activation of Snail and Serpent, among transcriptional repressors down regulates genes encoding for E-cadherin, claudin and occludin [18-20]. In addition Srp represses Crb apical polarity gene leading to redistribution of E-cadherin and Snail represses Crumbs3. Zeb1, Crumbs3 and Lgl2 interact. Total EMT can be executed by Snail even through the activation of matrix metalloproteinases. SNAI1 and SNAI2 are key inducers of EMT in gastrulating mouse [21]. Snail1 is prevalent on Snail2 as deletion of Snail2 in mice shows no EMT failure [22]. Schemes describing signaling pathways of EMT can be found in the major publication of Lim and Thierry [23]. EMT failure can be involved in embryological pathology. In this way, EMT seems to be implicated in cleft palate defect. The latter is one of the most common human congenital anomalies affecting around one case in 500–2500 live births. During palatal fusion, the midline epithelial seam between the palatal shelves degrades to achieve mesenchymal confluence. Fusion of the two palate shelves is a process involving cell death, adhesion and EMT. It implies EMT as a regulator of palatal fusion. The main inductor of this transition is TGFβ3 able to activate key EMT transcription factors like Lef1, Twist and Snail1. To support this hypothesis it was demonstrated that TGFβ3 null mice develop cleft palate [24]. Among other embryological pathologies issue from EMT deregulation, congenital heart defects can be suspected. Valvuloseptal endocardial cushion tissue arises from endothelial cells through a phenomenon called endothelial mesenchymal transition. The latter is mainly regulated by bone morphogenetic protein (BMP), TGFβ and mesenchymal status (EMT) that are essential area of medical research [25].

### Wound healing and EMT

EMT has a major role in wound healing and can explain some of its pathological aspects. EMT is mediated by inflammatory cells and fibroblasts. These cells secrete inflammatory molecules able to interact with proteins of ECM like collagens, laminins, elastin, and tenacins. [26]. Tissue wound healing evolves in three phases: inflammatory, proliferative and maturation phases. The aim of inflammation is to limit tissue damage through phagocytosis. The second phase leads to formation of granulation tissue, angiogenesis, deposition of new ECM and then re-epithelialization. The key step of wound healing is re-epithelialization. Keratinocytes become actively moving cells from the edges to the hole of the wound. Normally the epithelial layer of keratinocytes goes through differentiation of progenitor cells until cell death. This process causes the formation of the epidermis outer layer (cytokeratin skeleton and lipids mixture). This mechanical and hydration barrier protects the underlying tissue. The re-epithelialization is sustained by conversion of cells from sedentary state to the migratory one. This is due to EMT which is essential for wound repair. This modification of cellular phenotype is clearly profitable opposite to changes that occur in a tumor. Comparison of cancer and re-epithelialization EMT has clinical implications. Effectively it can give rise to conflict between cancer therapy and wound healing [27].

TGFβ is a major cytokine inducing EMT and also has other implications in wound healing. Moreover different growth factors can play a role in the EMT process. They include: hepatocyte growth factor, epidermal growth factor, insulin-like growth factor, connective tissue growth factor, tumor necrosis factor alpha, and fibroblast growth factor [8]. High levels of TGFβ have been detected in granulation tissue from healing thermal burn wounds and correlatively there was high expression of TGFβ receptors in fibroblasts involved in wound repair. The up-regulation of TGFβ can exceed its goal and leads to hypertrophic scars [28].

Osteopontin (OPN), a glycoprotein also named Secreted Phosphoprotein 1 has been implicated in 3 types (EMT associated with migration of cancer cells (metastasis) is referred as Type III. EMT process ongoing in embryogenesis is named Type I and Type II is linked to regeneration/fibrosis). OPN is able to bind different integrin receptors and several transcription factors regulated by TGFβ sustain the expression of OPN. Thus, OPN seems to play a central role in TGFβ-dependent processes and is involved in TGFβ dependent EMT [29].

### Fibrosis and EMT

The best fibrosis model depicted in clinical pathology is renal interstitial fibrosis. It is a progressive and lethal disease due to different grounds like urinary tract obstruction, chronic inflammation and diabetes [30]. EMT plays a key role in the development of renal tubular fibrosis and synthesis of extracellular matrix. Pathways of this pathological EMT are studied by numerous laboratories as new therapies could target it and be opposed to its progression. TGFβ upstream regulates many pathways. Among them are included Smad as well as MAPK-PI3k signaling pathways, TGFβ receptor kinase phosphorylates Smad 2 and 3. The activated latter interact with Smad4 that undergoes nucleus translocation, thus regulating transcription TGFβ target genes. TGFβ/Smad3 regulation seems to be essential in pathological fibroses [31].

Renal tubulointerstitial fibrosis leads to end-stage renal failure [32]. This process associates ECM deposition, inflammatory cells infiltration, fibroblasts accumulation with loss of tubular epithelial cells. The pathology is sustained by EMT targeting tubular cells. The latter acquire the classical markers of this pathway. The major growth factor driving the transformation is TGFβ. The reversion of EMT reduces fibroblasts proliferation and deposition of ECM in the cortical interstitium. Thus, the best choice to prevent progressive renal tubulointerstitial fibrosis is to regulate EMT [33]. Activation of hedgehog signaling that induces TGFβ expression has profibrogenic effects [34]. This pathway is activated by binding of the ligands including sonic hedgehog (Shh) to its membrane receptor patched 1 (Ptch1). Transduction by Smoothened (Smo), leads to translocation of the transcription factor Gli1 to the nucleus. This activation of hedgehog induces fibrogenesis. EMT in renal fibrosis has been debated [35-37]. Inoue et al. demonstrated that disease models and murine strains used in experimental conditions have to be taken into account to explain reported discrepancies [38]. Finally in a review, Galichon and Hertig showed the role of EMT markers in the diagnosis and prognosis of kidney failures [39]. They indicated that among EMT markers used in immunohistochemistry, the best could be simultaneously vimentin and β-catenin. At the opposite they excluded the fibroblast-specific protein (FSP1) and E-cadherin. They reported their study on renal allograft: three months after transplantation, vimentin and β-catenin had prognostic value and were associated with a more rapid progression towards graft interstitial fibrosis and decrease in renal function at twelve months. This paper is a proof that EMT research can be translated to clinical applications [40].

Recent publications of Leask et al. demonstrated that TGFβ is able to promote tissue repair and fibrosis trough the noncanonical focal adhesion kinase (FAK) pathway. FAK is implicated in myofibroblast differentiation. Thus acting on FAK pathway could be a major point to treat fibrosis disease. In a similar way excessive scarring could benefit from the same drugs [41,42].

### Cancer and EMT

The major headline of EMT in clinical application is related to cancer disease. Effectively there is a close link between EMT, circulating tumor cells (CTC) and metastasis. EMT endows tumor cells with new features, chemo and radiotherapy resistances. Thus, EMT is a major target to break the deleterious cycle of cancer. From the primary tumor, some epithelial cells can lose their cell-cell adhesion and become motile and invasive mesenchymal cells. These cells invade the ECM and migrate along a newly formed matrix of fibronectin and type I collagen [8]. They can move as single cells or be part of a collective migration: cell clusters. The latter made of cells with mixed phenotype (epithelial-mesenchymal) can avoid anoikis and lead more easily than single cells to metastasis [43]. Thus these results suggest that mesenchymal cells can protect from anoikis epithelial cells included in a cluster. Shed cells cross the ECM to reach vessels (intravasation) and by extravasation they colonize a distant organ in a specific niche. Then they stay as dormant tumor cells, micrometastasis or grow as a macrometastasis. The fate of such a new localization is depending on the mesenchymal epithelial transition (MET) which is the reverse way of EMT. We will review the different steps of this phenomenon.

From an epithelial tumor, cancer cells can reach vessels leading to CTCs. Many factors induce shedding of cancer cells. Transcription factors acting on gene expression are able to promote loss of cell-cell adhesions. As a result there is a shift in cytoskeletal anatomy and a change from epithelial morphology to the mesenchymal one. The EMT switch is on a signaling pathway dependence of TGFβ, BMP, Wnt–β-catenin, Notch, Hedgehog, and receptor tyrosine kinases. Many studies reported the role of several micro RNAs in the regulation of EMT and their interactions with ZEB1 and ZEB2 [44]. Izumchenko et al. demonstrated that the role of microRNA network on EMT-associated kinase switch [45]. Studies on EMT and micro RNA relations are a hot field now and would deserve a specific review. Moreover abnormal cancer epigenome is also implicated in control of EMT and stemness. Epigenetic deregulation evidently has a role in cancer that can be targeted in clinical trials [46].

The major stimulus able to activate the TGFβ pathway is hypoxia acting through HIF1α. The epithelial cobblestone growth pattern is held together by cell adhesion molecules (claudins and E-cadherin). The basal membrane anchors epithelial cells, through hemidesmosomes, to the ECM and provide their apical-basal polarity. Hallmarks of EMT are decreased expression of E-cadherin, tight junction proteins (ZO-1 and occludin) and cytokeratins while mesenchymal markers are overexpressed (vimentin, N-cadherin). Individual motile and shape spindle cells enter the ECM to reach vessels. This scheme is not as simple as described. Effectively a continuum of transformation from epithelial to mesenchymal cells has been suggested [47]. Moreover in two recent publications Jolly et al. proposed a mathematical model related to the process evolution [48,49]. They described the hybrid phenotype (epithelial and simultaneously mesenchymal) that gains likelihood stemness. This model could define the characteristics of cell clusters which are found among CTCs. Cancer stem cells have particular properties as they have the capacity to both self renew and differentiate into non stem tumor cells. A closed relation between induction of EMT and endowing of stemness characteristic has been demonstrated [46,50,51]. These kinds of hybrid cells seem likely to support micrometastasis and or relapses. In a recent publication, Ilie et al. demonstrated that cluster of hybrid cells are evidenced in the blood of patients with obstructive bronchopathy, at least 3 years before a primary lung tumor can be detected [52]. Aceto et al. demonstrated that CTC clusters are 50 fold more metastatic than single CTCs [43]. All these published results lead to the hypothesis that the major target to avoid relapse and metastasis in cancer are the hybrid phenotype (epithelial and mesenchymal) cells. Another therapeutic opportunity would be to interact on the reversion of EMT: MET. Effectively the major result of EMT is extravasation of CTCs into ectopic organs. After this step, cancer cells must survive in the adverse environment of organ parenchyma. There are new evidences that EMT is not irreversible and that reexpression of adhesion molecules due to MET promote survival and proliferation of cancer cells. One major factor of this reverse process seems to be the transcription factor MYB [53]. Moreover Ocana et al. showed that Twist downregulation favors metastasis formation. However silencing Twist alone is not sufficient to induce metastasis in the presence of PRRX1. PRRX1 loss is sufficient to reverse EMT even in the presence of other EMT inducers such as Twist1. Thus downregulation of PRRX1 leads to MET which goes along with acquisition of stem cell properties and increase of intermediary cell phenotype (epithelioid-mesenchymal) proliferation [54]. Targeting cells having EMT and cancer stem cell features appears a difficult task as normal stem cells share many identical characteristics. However Kreso et al. described a new therapeutic way to downregulate the BMI1-related self renewal without alteration of normal stem cells [55].

Many authors have compared EMT in different pathophysiological conditions. They assess similarities and discrepancies in protein expression and or signaling pathways in cancer and wound healing [56-58]. The CCN protein family interacts with integrins leading to release of growth factors, cytokines and matrix metalloproteinases. CCN2 and CCN4 are specifically up-regulated during wound healing while CCN3 and CCN5 are down-regulated [59-61]. The spectrum of CCN proteins could be one of the discrepancies between EMT of wound healing and cancer. Effectively, CCN1 and CCN6 have been characterized to have tumor promoting activity [62-64]. Both the Ras/ERK/MAPK pathway [65,66] and the PI3K/Akt/mTOR axis [67,68] are used in wound healing and cancer EMT. The discrepancy is rather based on transcription factor activity. Thus while Slug activity is upregulated in both wounded epithelium and in tumor cells [69-74], Snail has not been described as a major player during wound healing [75-80]. Moreover Zeb1, Ets-1, and FoxC2 seem to be an hallmark of cancer EMT. As both types of EMT share many similar signaling pathways, it is difficult to develop therapies targeting solely cancer EMT or wound healing EMT.

## Conclusion

EMT is a central physiological process for homeostasis and health of live beings. When shapely, fine tuned, during embryo development it leads to a normal anatomical body. The least failure of its regulating pathways sustained embryological defects. In a fully developed organism, when EMT is perverted, its activation is accountable for pathological situations as demonstrated in cancer and fibrosis diseases. EMT is still a beneficial way when acting in repair wounds. Nevertheless if we compared wound healing and cancer growth, we can consider cancer growth as a wound healing process that goes over its aim. Such opposing roles underline the difficulties to develop EMT drugs. Many therapies have been proposed to act on the receptors and/or signaling pathways that give rise to EMT. As mechanisms between cancer EMT and wound healing are shared, a conflict can rise between therapy of cancer and promotion of wound healing [27]. This review underlines the complexity of pharmacological improvements as EMT has conflicting aims according to its role in the targeted pathologies: fibrosis, wound healing, cancers.

## Abbreviations

EMT: Epithelial mesenchymal transition
MET: Mesenchymal epithelial transition
CTC: Circulating tumor cell
ECM: Extracellular matrix.
